# P-182. Chronic Infection Screening in Immigrant Patients Recently Diagnosed with Cancer

**DOI:** 10.1093/ofid/ofaf695.406

**Published:** 2026-01-11

**Authors:** Kelly Hallowell, Abdulsabur Sanni, Megan Shaughnessy

**Affiliations:** University of Minnesota, Minneapolis, MN; University of Minnesota, Minneapolis, MN; Hennepin Healthcare System, Minneapolis, Minnesota

## Abstract

**Background:**

Immigrants face different challenges in the setting of immunosuppression, as they may harbor chronic parasitic infections that can reactivate and/or worsen. Hennepin Healthcare System (HHS) does not have a protocol for infection screening prior to chemotherapy or immunotherapy for chronic parasitic infections such as *S. stercoralis* and *T. cruzi* or other chronic infections that can be exacerbated by immunosuppression such as HIV and *M. tuberculosis*.

**Figure:**
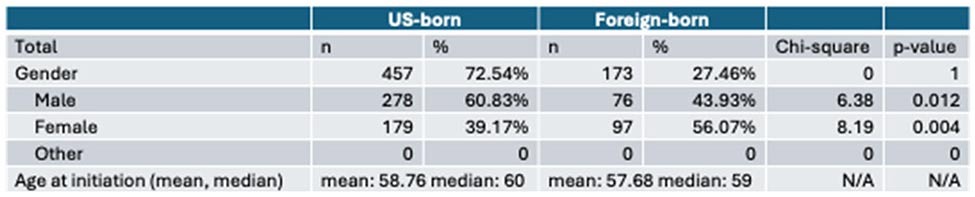
Patient Demographics The demographic information of the patients included in the analysis.

**Figure:**
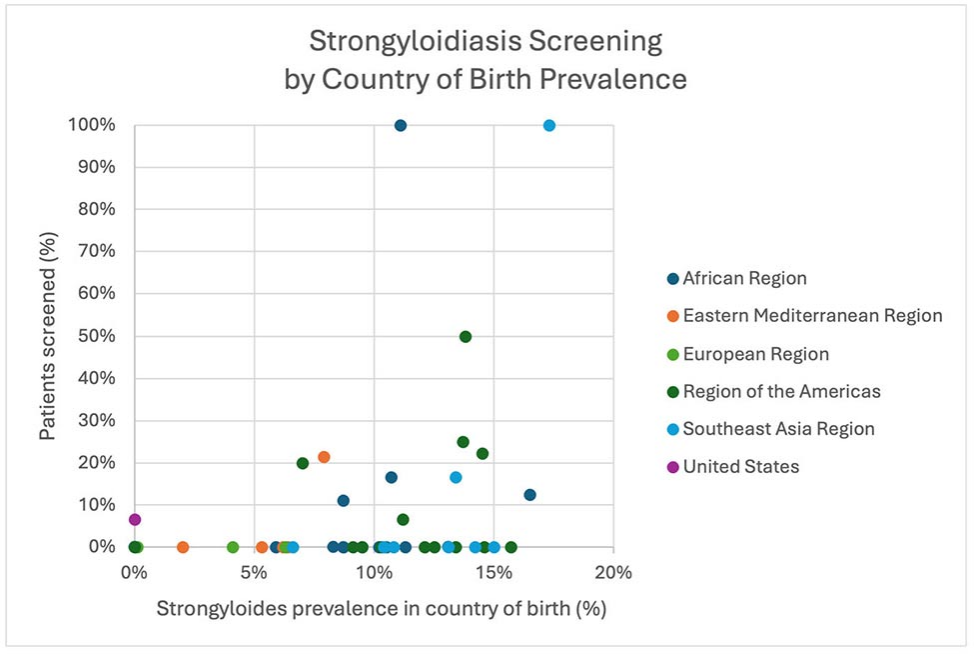
The percent of patients screened for strongyloidiasis prior to initiation of cancer treatment plotted against the estimated prevalence in their country of birth.

**Methods:**

A cross-sectional study of adult patients newly diagnosed with cancer who received treatment through HHS was performed from 1/1/2016-12/21/2021 through queries of the HHS Cancer Center database and Epic electronic medical record (EMR) evaluating for lab results for each disease ever prior to the day of initiation of chemotherapy and/or immunotherapy. A total of 1475 patient records newly diagnosed with cancer were reviewed and 630 patients met inclusion and exclusion criteria.

**Figure:**
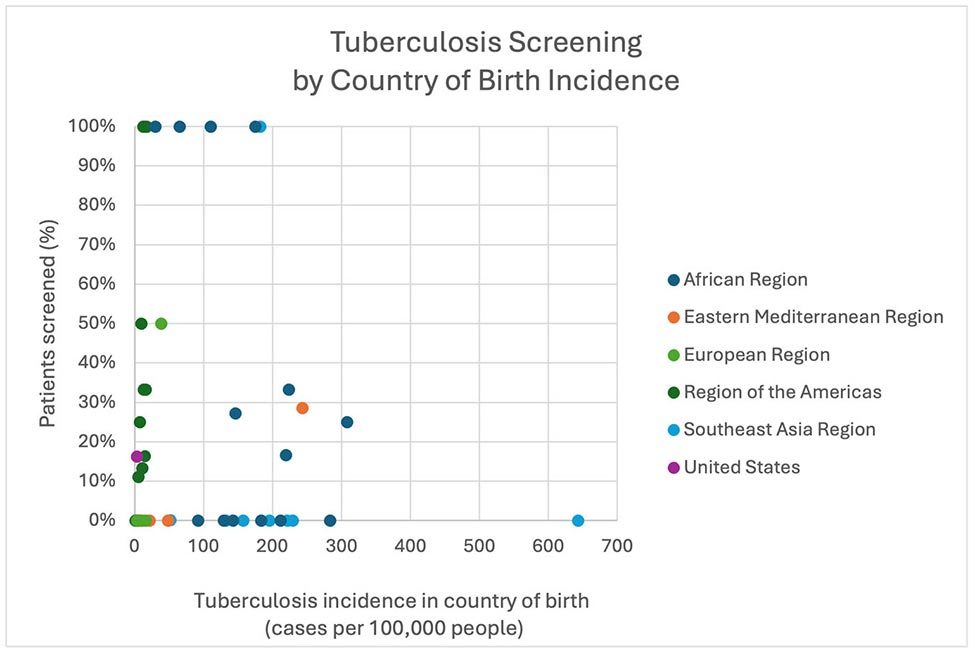
The percent of patients screened for tuberculosis prior to initiation of cancer treatment plotted against the case incidence per 100,000 people in 2023 in their country of birth.

**Figure:**
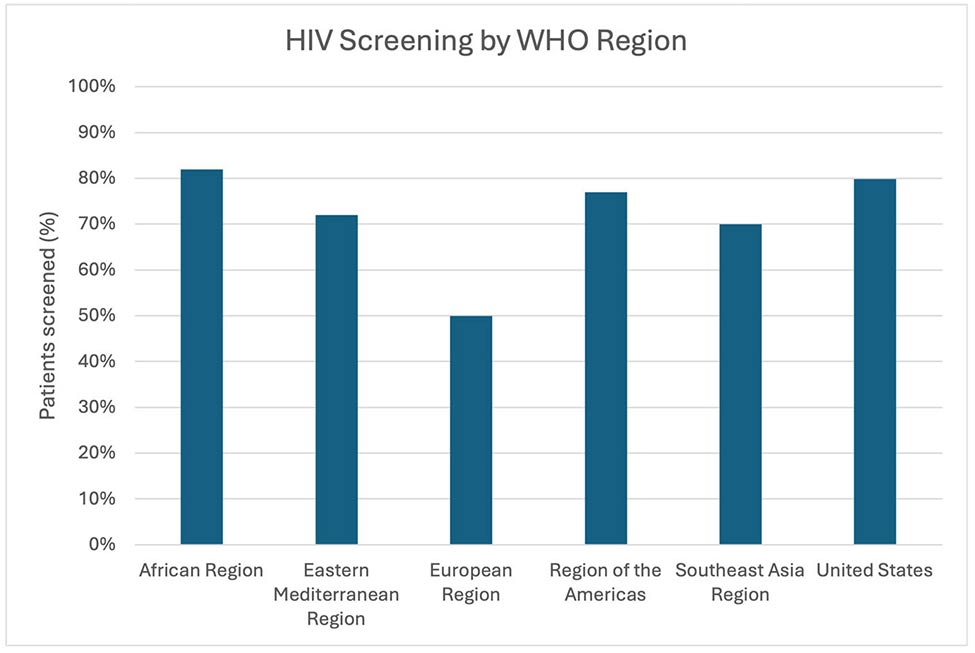
The percentage of patients screened for HIV prior to initiation of cancer treatment grouped by WHO region with US-born patients screening listed separately.

**Results:**

Prior to the administration of chemotherapy and/or immunotherapy, 79.89% of US-born participants compared to 92.47% of non-US-born participants were screened for HIV (**χ**^2^ = 1.99, p = .16), 20.96% of US-born participants compared to 21.92% of non-US-born participants were screened for tuberculosis (**χ**^2^ = 0.048, p = .83), and 8.50% of US-born participants compared to 16.44% of non-US-born participants were screened for strongyloidiasis (**χ**^2^ = 6.02, p = . 014). No patients were screened for *T. cruzi*. 58.21% of patients in the US-born group died within the study period compared to 32.95% of non-US-born patients after initiation of treatment within the study period (**χ**^2^ = 31.04, p = < 0.0001).

**Conclusion:**

Rates of screening for chronic infectious diseases with higher US prevalence were similar between groups. Within the immigrant groups, no patterns emerged based on reported rates of prevalence in country of birth although individuals per country were low. This study was limited by access to only laboratory results in the HHS EMR and did not delineate screening by reactivation risk of the chemotherapy and/or immunotherapy used. This work is being used to design screening guidance for oncology providers based on country of birth prior to cancer treatment.

**Disclosures:**

All Authors: No reported disclosures

